# Function estimation: Quantifying individual differences of hand-drawn functions

**DOI:** 10.3758/s13421-024-01598-5

**Published:** 2024-06-28

**Authors:** Daniel R. Little, Richard M. Shiffrin, Simon M. Laham

**Affiliations:** 1 Melbourne School of Psychological Sciences, The University of Melbourne, Parkville, VIC 3010 Australia; 2 Psychological and Brain Sciences, Indiana University, Bloomington, IN USA

**Keywords:** Graphical perception, Gaussian processes, Individual differences

## Abstract

Graphical perception is an important part of the scientific endeavour, and the interpretation of graphical information is increasingly important among educated consumers of popular media, who are often presented with graphs of data in support of different policy positions. However, graphs are multidimensional and data in graphs are comprised not only of overall global trends but also local perturbations. We presented a novel function estimation task in which scatterplots of noisy data that varied in the number of data points, the scale of the data, and the true generating function were shown to observers. 170 psychology undergraduates with mixed experience of mathematical functions were asked to draw the function that they believe generated the data. Our results indicated not only a general influence of various aspects of the presented graph (e.g., increasing the number of data points results in smoother generated functions) but also clear individual differences, with some observers tending to generate functions that track the local changes in the data and others following global trends in the data.

## Introduction

Since William Playfair introduced the convention in 1786 (Friendly & Denis, 2005), science has involved the examination and interpretation of graphical data. In papers, conference talks, and lectures, data are typically presented along with theoretical predictions, usually lines that capture qualitative trends or average predictions of the causal generating function. When viewed alongside predictions, our assimilation and confirmation biases can lead us to believe that the presented data generally fit with the predictions. The influence of the expectation set up by the theoretical predictions is such that it is as if we mentally reorganize the data to better match the predictions. But what happens when we view data in that absence of any prediction? How are our inferences shaped by perceptual and cognitive biases (e.g., toward simplicity; Lombrozo, 2007)?

**Fig. 1:**
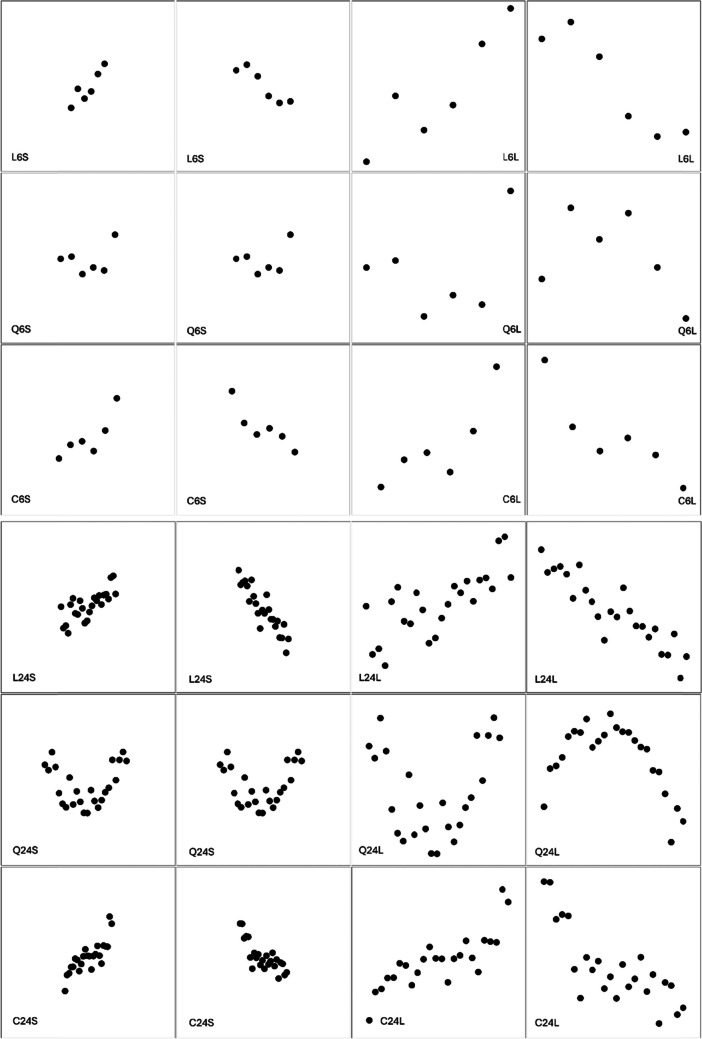
*S*catterplots used in our function estimation task. Scatterplots vary in the number of data points, whether the scale of the figure is zoomed in or zoomed out, and the underlying generating function (linear – top row, quadratic – middle row, or cubic – bottom row). All plots were shown in both zoomed in (first and second column) and zoomed out (third and fourth column) formats. Problems are indexed by Generating function (L = Linear, Q = Quadratic, C = Cubic), Number of data points, and Scale (S = Small, L = Large)

We explore these questions using a little examined task that we term *function estimation*. Participants are asked to examine scatterplots of noisy data and draw the function that they believe generated the data. Examples are shown in Fig. 1; we invite the reader to mentally estimate the function that generated each dataset. In our initial pilot tests, and to foreshadow the present results, in our current study, we find large individual differences in how people estimate functions; some generate smooth, polynomial-like functions that capture the global trends in the data, but others generate functions that track the local fluctuations in the data. We first review several findings that provide clues to the locus of these differences.

Studies of statistical estimation from graphs highlight several different perceptual features that observers use to drive inference. These features fall into two broad categories of attributes that can be attended and that influence the inferences people make: *local* features or dimensions of the data whose value is specific to single points, pairs, or small groups, or *global* features, which reference overall trends. Studies of hierarchical stimulus perception (e.g., a large letter E composed of small letter Ss) suggests a bias toward global features (Kimchi, 1988; Navon, 1977); however, several perceptual influences modulate this bias and there are large individual differences in the expression of this bias. A brief survey of categorization and function learning reveals that individual differences in inference tasks are common and related to differences in selective attention (Bartlema et al., 2014; Craig & Lewandowsky, 2012; Delosh et al., 1997; Erickson & Kruschke, 1998; Kalish et al., 2004; Lewandowsky & Kirsner, 2000; Lewandowsky et al., 2002, 2006; Little & Lewandowsky, 2009; Rouder & Ratcliff, 2004; Sewell & Lewandowsky, 2011; Yang & Lewandowsky, 2003, 2004) or to individual differences in knowledge and expertise (Lewandowsky et al., 2007; Medin et al., 1997; Wing et al., 2022). Given the subjectivity of our function estimation task and the multidimensional nature of graphical data, individual differences are to be expected. Nonetheless, the nature and extent of those differences is a novel and interesting question given the importance of graphical inference in science and in general.

### Graphical perception

Graphical perception is a crucial part of science; further, the interpretation of graphical data is increasingly important among the educated consumers of popular media, who are often presented graphs of data in support of some position. Graphical displays can present the true relationship between two variables more accurately than summary statistics (e.g., correlations; Cleveland et al., 1982). However, care must be taken in the presentation of graphical information to avoid confusion due to visual illusions or biases in visual perception (Franconeri et al., 2021). That is, the interpretation of graphical information is an inherently visual activity and draws on the properties of the visual attention system (Franconeri, 2021; Michal & Franconeri, 2017). Visual properties of graphs interact with conceptual knowledge to formulate a causal understanding of the presented information.

Here we examine function estimation by asking observers to draw their best estimate of the function that generated a presented scatterplot. Scatterplots represent between 70 and 80% of scientific graphs (Friendly & Denis, 2005; Tufte, 1983); consequently, this task taps a very common quantitative inference. At first blush, we might expect function estimation to demonstrate a bias toward simplicity in line with rational Bayesian reasoning (i.e., see e.g., Chater & Vitanyi, 2003; Little & Shiffrin, 2009). Psychologically, quantitative inferences fulfil the same role that models do in science; both allow for the summarization of existing data, increase our understanding of the underlying causal mechanisms, and allow for extrapolation and prediction beyond the given data (Simon, 2000). Like scientific models, function estimation, like other forms of explanation, should trade off fit and complexity. For instance, simple verbal explanations are assigned higher probabilities than complex verbal explanations in the absence of any information about how well the explanation fits the data (Lombrozo, 2006, 2007). When evaluated in light of existing data, the simplicity of an explanation trades off against how well the explanation fits the data, in a manner consistent with updating explanations in a Bayesian framework (Lombrozo, 2007).

On the other hand, graphical data must be filtered through perception, and it is unlikely that the information used for psychological inference is the same as what one might input to statistical inference. The implication is that although the mechanisms underlying inference might be the same in both statistical and mental inference, the outcome might be very different due to differences in the *data* that are to be explained. In other words, perceptual biases may lead to different outcomes in psychological inferences because these biases may provide differential weightings to certain data features, weightings that will differ from pure statistical inference (which is free from perceptual biases). Consequently, mental function estimation may not be simply a matter of statistical curve-fitting. There are also other factors, such as comprehension of mathematical function knowledge and prior experience with functions that should affect mental function estimation.

### Perceptual influences on graphical inference

Several studies have demonstrated that perceptual aspects of graphs influence mental inference (for reviews, see Franconeri et al., 2021; Lewandowsky & Spence, 1989). For instance, in an early study of function estimation, Mosteller et al., (1981; see also Collyer et al., 1990) presented subjects with scatterplots generated from a linear function with small or large additive noise. Participants drew their best estimate of a linear function. The inferred functions were much closer to the principal component axis than the best linear regression line. That is, the functions were drawn such that they tended to minimize errors on both the x and y dimensions rather than on the y dimension alone (as in regression). Although early studies had shown that participants could judge to a close approximation when a regression line was at its maximum likelihood value (Finney, 1951), hand-estimation of a function was influenced by the location of the perceived major axis of the ellipse of noisy data points.

Participants are more accurate at estimating correlations from noisy data if the scale of the axes relative to the data is decreased (i.e., when the data are shown as a small cluster rather than a larger cluster on the same axis; Cleveland et al., 1982; Collyer et al., 1990). The primary perceptual dimensions used to judge correlation are elongation (i.e., the ratio of the major and minor axis) and density (i.e., how close the data points are in the graph; Boynton, 2000). Increased elongation increases the correlation estimates for a given level of perceived density but decreasing density by increasing the scale of the data results in lower correlation estimates. Ultimately, these results indicate that there are several available visual cues that affect inference from noisy data.

### Local predictors of graphical inference

In the present study, we’re interested in function estimation rather than estimation of a correlation, and as a consequence, we ask about graphs generated from a number of different function types (e.g., linear, quadratic, and cubic) and not solely linear functions (as in previous studies). In the absence of additive noise, inferring the generating function, within some interpolated region, is equivalent to inferring the line of curvature on which all of the data points lie (i.e., estimating collinearity). Feldman (1997; see also Feldman, 1993, 1996) introduced a model in which collinearity was evaluated by considering the probability that each successive angle between pairs of dots was generated from a Gaussian distribution such that small angles were highly probable from collinear points but large angles likely indicated that the points did not fall on the same line. In other words, there is a prior expectation that collinear points have small successive angles. Estimations of collinearity are largely consistent with a Bayesian analysis formulated to represent this bias, and similar models have been proposed to explain other visual phenomena such as contour detection (Field et al., 1993; Hess & Field, 1999) or edge detection (Geisler et al., 2001). The importance of local elements in collinearity or contour judgements was demonstrated empirically by Hon et al., (1997; see also Warren et al., 2002, 2004), who had observers adjust an aberrant point to lie on a mentally interpolated contour inferred from a small number of dots. By systematically varying perturbations in the dots and measuring the influence of the perturbation on the adjusted point, Hon et al. were able to show that the interpolated contour was only influenced by the points closest to the to-be-adjusted points (i.e., two-points on either side).

In these curve interpolation experiments (Feldman, 1993, 1996, 1997; Hon et al., 1997; Warren et al., 2002, 2004), there was no noise added to the data points, and furthermore, only a small number of data points (e.g., 3–8) were presented. Consequently, the angle between pairwise points was a highly valid local predictor of collinearity. Even when extra noisy points were added around the contour points to provide a contextual background such that points perturbed far from the contour could be interpreted as noise, interpolation still only relied on the points closest to the adjustable point (Hon et al., 1997; Experiment 3).

In the current experiment, we examine functions with both a small and a large number of data points, different generating function types, and considerable amounts of noise. This leaves several different dimensions available for guiding inference. On the one hand, reliance on relatively local features could lead to functions that tend to track each fluctuation in the data. On the other hand, due to the increased level of additive noise, participants might instead use more global features, such as the overall trend of the points resulting in smooth, polynomial-like functions. To some extent, commonalities in perception of dot grouping might lead to a high degree of consistency in the generated functions in the same way that commonalities in visual perception drive similar constellation groupings across cultures (Kelly et al., 2024; Kemp et al., 2022a, 2022b). On the other hand, the multidimensional nature of graphs coupled with differences in mathematical knowledge lead us to expect different individuals in attention to different aspects of the graphs resulting in different estimated functions. In particular, based on our pilot data, we expect some individuals to focus more on the global aspects of the data, and others to focus on the local aspects.

### Individual differences

Individual differences are apparent in the results of many cognitive tasks, especially when the tasks are relatively open-ended with no explicit correct answer. These differences are often driven by differences in selective attention (i.e., the relative weighting given to stimulus dimensions; Nosofsky, 1986; see for instance, Little & Lewandowsky, 2009; Yang & Lewandowsky, 2004). For accurate performance, valid cues should be weighted more than nonvalid cues; although, salient cues trade off against valid cues in a straightforward manner (Kruschke & Johansen, 1999). Differences in attention can also be driven by differences in knowledge (cf. Medin et al., 1997). Through learning, observers typically weight cues optimally (i.e., to maximize accuracy; Nosofsky, 1986; Vanpaemel & Lee, 2012), but individual differences in attention are prevalent (see, e.g., Bartlema et al., 2014; Erickson & Kruschke, 1998; Rouder & Ratcliff, 2004). Bartlema et al. (2014) analysed the results of several categorization tasks (e.g., Kruschke, 1993 and Nosofsky, 1986) and showed that while some participants attended mostly to one of the dimensions, another group attended mostly to the other dimension and a third group largely guessed the answer. Consequently, even when one might expect invariant behaviour across subjects, individual differences may be obscured in the aggregate (Estes, 1956; Lee & Webb, 2005; Liew et al., 2016; Navarro et al., 2006). Individual differences are also found in function learning, which is particularly relevant for the current inquiry.

### Function learning

In function learning, a continuous response is predicted on the basis of a continuous stimulus (DeLosh et al., 1997; Kalish et al., 2004; Koh & Meyer, 1991; Lewandowsky et al., 2002). Different values of the stimulus are presented on each trial; with feedback, people can learn complex relations with varying degrees of success. Random stimulus–response pairings are difficult to learn (Carroll, 1963; Fitts & Deininger, 1954), but more coherent functions, such as positive and negative linear functions or parabolic functions, can be learned (Bjorkman, 1965; Sheets & Miller, 1974) with linear relations being easier to learn than non-linear relations (Bjorkman, 1965; Brehmer et al., 1974; Sheets & Miller, 1974).

A defining attribute of function learning is extrapolation, but people vary in how they extrapolate. Delosh et al. (1997) trained participants on a quadratic function and found that some extrapolated along the parabola, suggesting that they had inferred the underlying training function. Other participants, however, produced extrapolation points (i.e., y-values) that were close to the output value of the most similar input (i.e., the x-values), suggesting that they had simply memorised the underlying training values. This tendency to extrapolate in a smooth polynomial-like manner rather than based on similarity to nearby points seems to be a stable individual difference which predicts rule-use vs exemplar memory, respectively, in categorization (McDaniel et al., 2014).

The representations formed in function learning studies are determined in part by cognitive limitations on, and the processes of, attention, learning, and memory. For example, the relation between an input value and an output value presented at one point in time might be partially or wholly forgotten later, and possibly distorted through inference in the direction of simplicity. As another example, extreme points might be attended and remembered best, leading to a different set of distortions. In the current paper, we present an experimental method for studying function representations in which all the data (i.e., the training stimuli) are presented simultaneously. In this method, one should not have to parcel out the effects due to faulty memory for the data points on the function or inadequate learning of the function over time. Instead, we expect any individual differences that arise to reflect differences in attention to local or global elements of the graph or differences in an underlying predisposition toward either locally influenced, similarity-based functions, or smooth, polynomial-like functions.

### Function estimation

In the following studies, we presented observers with several scatterplots and asked participants to draw the function that they believed generated the data. In line with studies of correlation perception, we varied the number of points and varied the scale of the data (i.e., zoomed out or zoomed in). We additionally varied the true generating function (linear, quadratic, or cubic). Gaussian noise was added to each generated function.

Each of these manipulations are expected to affect the relative salience of the local perturbations in the plot versus the overall global trends. That is, although we expect individual differences based on the literature reviewed above, we also expect an effect of each of these manipulations; that is, some manipulations should constrain the types of functions that are generated (e.g., graphs with many data points and little noise should result in less individual variation than a data set with a small number of points and high noise). In other words, the extent of individual differences should depend on specific aspects of the data (cf. Peterson & Deary, 2006).

More specifically, we expect that in graphs with small numbers of points, observers might be influenced by interpolated contours between nearby points (cf. Feldman, 1997; Hon et al., 1997); consequently, tendencies toward functions that track local perturbations should emerge more in graphs with fewer points to a greater extent than in graphs with larger points. Likewise, at smaller scales, the global form of the data should be more evident, perhaps resulting in a greater tendency to follow the global trend in the data. At larger scales, local fluctuations in the data should be more salient, which should increase trends toward data tracking.

Our participants are all Australian university psychology undergraduates. It is common at Australian universities to enter the psychology major either from the Bachelor of Arts or Bachelor of Science; consequently, although the students are university students, the extent to which mathematics is emphasised in their degrees varies dramatically. Hence, the extent to which these scatterplots and functions have any relevance to their studies or everyday life varies. We would emphasize that this is also true in the case of the real-world issue of scientific visualization and interpretation. The presentation of scientific data is often arguably pitched at an educated public but one that has differential understanding of relevant concepts such as polynomial functions and variability (cf. Kahneman et al., 2021).

To foreshadow, in our analyses, we use Gaussian process regression (Rasmussen & Williams, 2006), which allows for the specification of the underlying functional form of the generated function using a specific type of kernel (e.g., a radial basis function kernel; Griffiths et al., 2009) with hyperparameters that can be tuned to capture functions that are smoother, reflecting global data trends, or fluctuate more, reflecting local data trends. After applying this analysis, we then use a nonparametric Bayesian clustering algorithm (Navarro et al., 2006) to capture individual differences in the latent parameters, which capture how people generate functions.

## Method

### Participants

One hundred and eighty University of Melbourne psychology students received course credit for participation. Data from three participants were excluded for failure to follow instructions, leaving 177 participants. Human testing was approved by the University of Melbourne Human Ethics Advisory Group.

### Procedure and design

The function estimation test was administered as part of a larger paper-based questionnaire. Additional unrelated components were presented after the function estimation questions and are not considered further. We presented 24 scatterplots (see examples in Fig. 1; the full set of graphs is shown in the Online Supplementary Material (OSM), which is available at https://github.com/knowlabUnimelb/FUNCTION_ESTIMATION). Two 7.5 cm × 7.5 cm graphs were presented per page with a 4-cm gap between them. Participants were instructed that each plot showed data from a different fictional scientific experiment. Participants were told to view each graph and to draw what they believed to be the true causal function. Exact instructions are shown in the OSM.

Scatterplots varied in three ways:

(i) the number of sampled points was small (N_d_ = 6) or large (N_d_ = 24).
(ii) The scale of the data was either large (see the top row of Fig. 1), such that the data took up the entire figure, or small (see the bottom row of Fig. 1), such that the data were presented centrally but only filled 40% of the total area. The small scale appeared as a “zoomed out” version of the large scale. The relative position of the points in the small- and large-scale sets was identical.
(iii) The function used to generate the data was a linear, quadratic, or cubic polynomial. Gaussian noise was added to the generating function to produce the 24 graphs. We refer to this as *stimulus* noise to keep it distinct from the noise variance parameter estimated by the Gaussian process regression, which we describe next.

### Data analysis

To allow the hand-drawn functions to be analysed, we scanned each of the drawings creating a digital.jpg image. Digital images were loaded into Data Thief (Tummers, 2006), a software program that allows manual extraction of numerical value from figures. All axes were scaled to range between -1 and 1. The software returns a set of finely sampled points tracking the drawn function. One of the authors (DL) manually viewed each of these extracted functions, systematically correcting for any errors by either concatenating parts of functions that were not extracted as a single function or, in rare occasions, deleting overlap where multiple lines were drawn (e.g., where the participant backtracked along the x-axis). Twenty participants exhibited odd or missing data for some functions; any usable functions were maintained for these participants. We then down-sampled the final processed responses to a set of 40 evenly spaced points in the range of the x-axis.

### Gaussian process regression

To capture individual differences, we used Gaussian process regression (GPR), which is flexible enough to capture both data-tracking functions and smoother more polynomial-like functions. We fit a highly flexible regression model to each observer’s generated functions and then examined the parameters of that model in order to classify each function as either locally or globally influenced. In this manner, we are using the GPR as a measurement model to differentiate the two primary types of responding identified in our preliminary visual inspection of the data.

As explained in more detail below, each Gaussian process requires specification of a *kernel*, which determines the form of the functions that the Gaussian process can generate. A kernel can be thought of as a way of specifying the similarity between two objects (cf. Jäkel et al., 2007); in this case, the kernel specifies the similarity between each point on the function and all other points on the function (Rasmussen & Williams, 2006). We adopted an exponential-weighting kernel (radial basis function) in which the similarity of each point to other points is, depending on the parameter values, either influenced only by nearby points at each x-value (i.e., local data tracking) or influenced by all the points in the graph (i.e., global trend estimation). Before describing this kernel in detail, we first briefly introduce GPR using the potentially more familiar polynomial functions.

The goal of regression is to find a relationship that maps input variables, X = x_1_,…,x_n_, to an output variable, y. Typically, this involves finding the value of some weight, $\beta$, on each input variable in X, assuming some additive Gaussian noise, $\varepsilon$:

1 $$y=\beta X+\varepsilon$$

Input variables might be different measures, dimensions, or basis function transformations (e.g., polynomial inputs: X = x^0^, x^1^,…,x^n^). Standard Bayesian regression requires specification of a prior over the $\beta$ s and the variance of the additive noise, ${\sigma }^{2}$, which in turn allows the posterior distribution of the weights and the noise variance to be computed via Bayes’ rule:

2 $$p\left(\beta ,{\sigma }^{2}\left|X,y\right.\right)=\frac{p\left(X,y\left|\beta ,{\sigma }^{2}\right.\right)p\left(\beta ,{\sigma }^{2}\right)}{\int p\left(X,y\left|\beta ,{\sigma }^{2}\right.\right)p\left(\beta ,{\sigma }^{2}\right)d\beta d{\sigma }^{2}}.$$

A Gaussian process is a generalization of a Gaussian distribution, which takes advantage of the fact that a multivariate Gaussian distribution or process conditioned on some dimension of X is also Gaussian (see Bishop, 2006, p. 85). In GPR, we conditionalize on the observed x and y-values to predict the most probable locations of a function at all points of interest on the x-axis (i.e., the entire range of the x-axis including any extrapolation and interpolation points). Like standard Bayesian regression, this requires specification of a prior, but in GPR this prior is over functions (making it a process rather than a distribution) and not over parameters. The prior is specified by establishing an expected mean and covariance for the functions. A common uninformative prior, which we also adopt here, is to assume that the prior mean function is 0 at all points. The covariance matrix, however, plays a more important role in that it determines the functional form (in smoothness and stationarity) of the Gaussian process.

The covariance matrix is created through the specification of a kernel, *k*, parameterized with hyperparameters, $\theta =\left[{\theta }_{1},...,{\theta }_{m}\right]$, which can be thought of as a measure of similarity between pairwise data points. For example, we could employ a polynomial-based kernel in which the similarity between two (possibly multidimensional) points, ${x}_{i}$ and ${x}_{j}$, is:

3 $$k\left({x}_{i},{x}_{j}\right)={\left({{x}_{i}}^{T}{x}_{j}+c\right)}^{d}$$

where the superscript *T* indicates the transpose operation, *c* is a constant, and *d* is the degree of the polynomial. A GPR analysis with this kernel produces analogous results to Bayesian polynomial regression. That is, the inferred functions all have an underlying polynomial form specified by the polynomial degree. Functions are then distributed as a Gaussian process with a specified mean and covariance:

4 $$f\left(x\right)\sim GP\left(m\left(x\right)=0,k\left(x,x^{\prime}\right)\right)$$

where $k\left(x,x^{\prime}\right)$ is computed over all pairs of training inputs.

#### Accommodating drawing data

In our present study, even functions that appear to be linear (or other well-executed polynomials) are likely to deviate from linearity in systematic ways due to biases introduced by hand movement. That is, even though an estimated function might look mostly linear, any slight curvature introduced by movement will reduce the likelihood that the estimated function was a linear polynomial when fit in a standard manner. One further reason for not adopting a polynomial kernel is that the data-tracking functions observed in our data can only be well fit using higher degree polynomial functions; however, given well-known biases toward simplicity, we do not believe that people are overfitting the data by estimating high-degree polynomials. Instead, it is more likely that the local features of the data influence responding such that nearby x-axis regions of the data are thought to result in similarly generated y-axis values. Consequently, instead of fitting a polynomial GPR, we adopted a kernel that can handle both globally and locally influenced functions. There are many other kernels that would have sufficed for this purpose; however, we adopted the *squared exponential* kernel, which is related to the Gaussian probability distribution (and is therefore often referred to as a Gaussian kernel or also the radial basis function; Griffiths et al., 2009) and also to the exponentially decreasing similarity functions incorporated into well-known models of categorization (e.g., GCM; Nosofsky, 1986) and has been previously used in a GPR analysis of function learning (Griffiths et al., 2009).

**Fig. 2:**
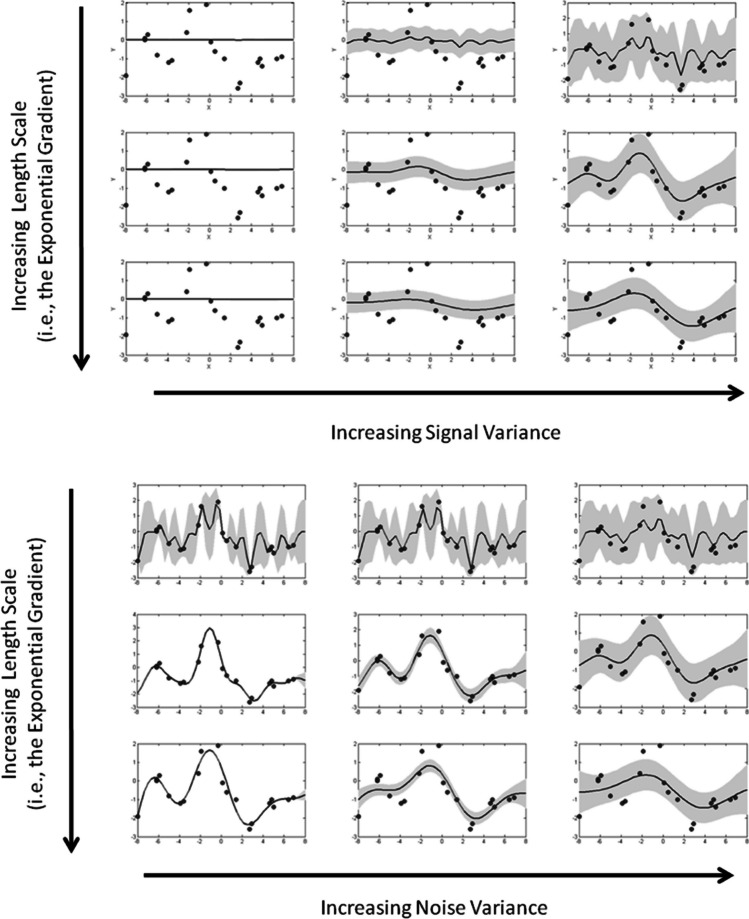
Effects of changing the hyperparameters of the radial basis function kernel on the predicted functions. The top set of panels shows the effects of increasing the signal variance parameter (left to right) along with the effects of increasing the length scale parameter (top to bottom). The bottom set of panels shows the effects of increasing the noise variance parameter (left to right) along with the effects of increasing the signal variance parameter (top to bottom). Note that the data in each figure is the same; changing the parameters changes the inferred generating function along with the estimated noise in the data

The *squared exponential* kernel evaluates similarity between two points as follows:

5 $$k\left({x}_{i},{x}_{j}\right)={\sigma }_{s}^{2}\text{exp}\left(-\frac{1}{2{\lambda }^{2}}{\left({x}_{i}-{x}_{j}\right)}^{2}\right)+{\sigma }_{n}^{2}{\delta }_{ij}$$

where the parameters, the *signal variance*, ${\sigma }_{s}^{2}$, the *length scale*, $\lambda$, and the *noise variance*, ${\sigma }_{n}^{2}$, change the shape of the underlying generating function in different ways. It is useful to refer to Fig. 2 to see the effects of varying these hyperparameters. The top nine panels of Fig. 2 show the effects of varying the length scale, $\lambda$, which is increased from the top row of panels to the bottom row of panels and the signal variance, ${\sigma }_{s}^{2}$ which is increased from the left to the right; *noise variance* is fixed at an intermediate value. Note that noise variance is distinct from stimulus variance in that the stimulus variance is noise added to the stimulus-generating function, whereas noise variance is a parameter in the Gaussian process kernel that informs the inferred generating function.

If we consider this kernel to be exponentially weighting the observed x-values near the to-be-predicted x-value, then the length scale parameter determines the width of the interval either side of a training data point that influences the current prediction. Smaller length scales lead to more complex functions, which tend to track the data more and are influenced more by local perturbations (see the top row of the top set of panels in Fig. 2) than larger length scales, which are more influenced by global trends in the data (see the bottom row of the top set of panels in Fig. 2). The signal variance parameters control the magnitude at which the observed data influence the predicted function. Smaller signal variance leads to more flat, horizontal functions (reflective of the 0 mean prior function); larger signal variance leads to functions that are more influenced by the data. Thus, at large signal variance the length scale parameter has more of an influence.

The bottom set of nine panels show the effects of varying the length scale (increasing from top to bottom) and the noise variance parameter (increasing from left to right) with signal variance fixed at an intermediate value. With increasing noise variance, the uncertainty in the underlying function increases, which is indicated in the figures by the increase in the grey region representing the 95% confidence intervals. Like the signal variance, the noise variance parameter moderates the effect of varying the length scale. Nevertheless, this kernel is able to produce both very smooth functions that follow the global trends in the data but also functions that track local changes in the data.

#### Fitting the model

Given a specific kernel, and using the conditional property of Gaussians, we compute the posterior predictive mean and variance across the entire response space (i.e., the x-axis), $\widehat{X}$, using the following equations:

6 $$\mu =k{\left(\widehat{X},X\right)}^{T}k{\left(X,X\right)}^{-1}y$$

and

7 $$\Sigma =k\left(\widehat{X},\widehat{X}\right)-k\left(\widehat{X},X\right)k{\left(X,X\right)}^{-1}k\left(X,\widehat{X}\right),$$

respectively (Rasmussen & Williams, 2006). We assume that the values of the response function at each queried point are distributed according to the predictive posterior distribution given above (i.e., $\widehat{y}\sim N\left(\mu ,\Sigma \right)$). To find the optimal values of the hyperparameters, we use gradient descent to minimize the log marginal likelihood, $\text{log}p\left(y|X,\theta \right),$ (Rasmussen & Williams, 2006, p. 112). The log marginal likelihood is given by:

8 $$\text{log }p\left(y\left|X,\theta \right.\right)=-\frac{1}{2}{y}^{T}{K}^{-1}y-\frac{1}{2}\text{log}\left|K\right|-\frac{n}{2}\text{log }2\pi$$

which can be seen to be the logarithm of the Gaussian probability density function, where *K* is $k\left(x,x^{\prime}\right)$.

Rasmussen and Williams (2006; p. 113) illustrate that the second term in the log marginal likelihood acts as a penalty on complexity, which for the squared-exponential kernel offsets the accuracy of the fit (computed in the first term) in the following way: As the length-scale increases and the functions become more influenced by the global-trends in the data, the fit decreases, but the penalty for complexity also decreases because the function becomes less complex. By contrast, for smaller length scales, the complexity penalty increases but so does the fit value. In this manner, the GPR trades off fit and complexity and can be treated as measuring the posterior model parameters that give the best estimated function for each response.

Note that we fit the model to the drawn response functions (after processing; see *Method*) rather than the stimulus scatterplots that were presented to observers. Our goal is to use the Gaussian process regression to capture individual differences in the drawn functions, not to provide a cognitive model of function estimation. Hence, we are using the Gaussian process regression as a measurement model to uncover parameters representing the complex array of functions that we observe in our data. We then attempt to characterize the qualitative individual differences by clustering the estimated parameters.

#### Parameter clustering

Having found the optimal parameters for each response function, we analysed individual differences by clustering those parameters using a nonparametric Bayesian clustering analysis. Navarro et al. (2006) introduced a method for identifying latent groups of individuals with qualitatively different types of performance. In this method, a Dirichlet process prior is set over the mixing weights between the likelihoods for each subset of individuals. This approach allows a potentially infinite number of latent groups to be determined; consequently, the number of clusters need not be specified in advance. The basic assumptions underlying the use of a Dirichlet process prior over the weights in a mixture model is that (a) the number of latent groups is unknown but can be estimated from the data if we assume that (b) the probability of joining a group is proportional to the size of the group, and that (c) the probability of forming a new group is proportional to a parameter,$\alpha$. The posterior distribution over the group assignments, *g*_*i*_ (i.e., the group index of data point *i*) can be estimated using Gibbs sampling with the following conditional probabilities (see Navarro et al., 2006):

9 $$p\left({g}_{i}=G\left|{g}_{-i},\alpha ,x\right.\right)\propto p\left({g}_{i}=G\left|{g}_{-i},\alpha \right.\right)p\left({y}_{i}\left|{g}_{i}\right.=G,{g}_{-i},{y}_{=i}\right)$$

The first term on the right-hand side is a Dirichlet Process prior given by:

10 $$p\left({g}_{i}=G\left|{g}_{-i}\right.,\alpha \right)\propto \left\{\begin{array}{cc}\frac{{n}_{G}}{n-1-\alpha }& \text{if }{n}_{G}>0,\\ \frac{\alpha }{n-1-\alpha }& \text{otherwise.}\end{array}\right.$$

The second term, the predictive likelihood of data point *I* given the other data points from group is the posterior probability of observing parameter value ${y}_{i}$ given the parameters already observed in group G. We assume that the predictive likelihood can be found by integrating over the parameters of the distribution of the group members. We assume that each group is a normal distribution with an inverse-Wishart distribution over the priors, which implies via conjugacy that the posterior over the group parameters is also a normal-inverse-Wishart distribution. Integrating over this posterior gives the predictive likelihood; this integral is a Student’s t-distribution, which we approximate as a Gaussian with a mean and covariance determined by the members of that group (see Sudderth, 2006, p. 47; the Dirichlet process clustering was conducted using a Matlab toolbox, available from https://github.com/jacobeisenstein/DPMM).

### Expert classification

For the cluster analysis, we had two independent raters classify each of the 24 functions from each observer as linear, quadratic, cubic, data-tracking, or unclassifiable. These classifications were then used to interpret clusters of parameters from the GPR analysis. Examples of each type of function were provided to each rater prior to rating. The correlation between raters was *r* = 0.81, *p* < 0.001.

## Results

In this section, we first present all the graphs along with the hand-drawn functions to illustrate the type of individual differences that arise in our data. Then we apply GPR, which offers a flexible way to capture the variety of functions drawn by individuals. We use the parameters estimated from the application of the Gaussian process to infer the influence of different graphical presentation factors (i.e., amount of data, scale, and generating function). Finally, we apply a clustering analysis to understand how the Gaussian process parameters are mapped to qualitatively different functions types (e.g., smooth polynomials vs. data tracking functions).

**Fig. 3:**
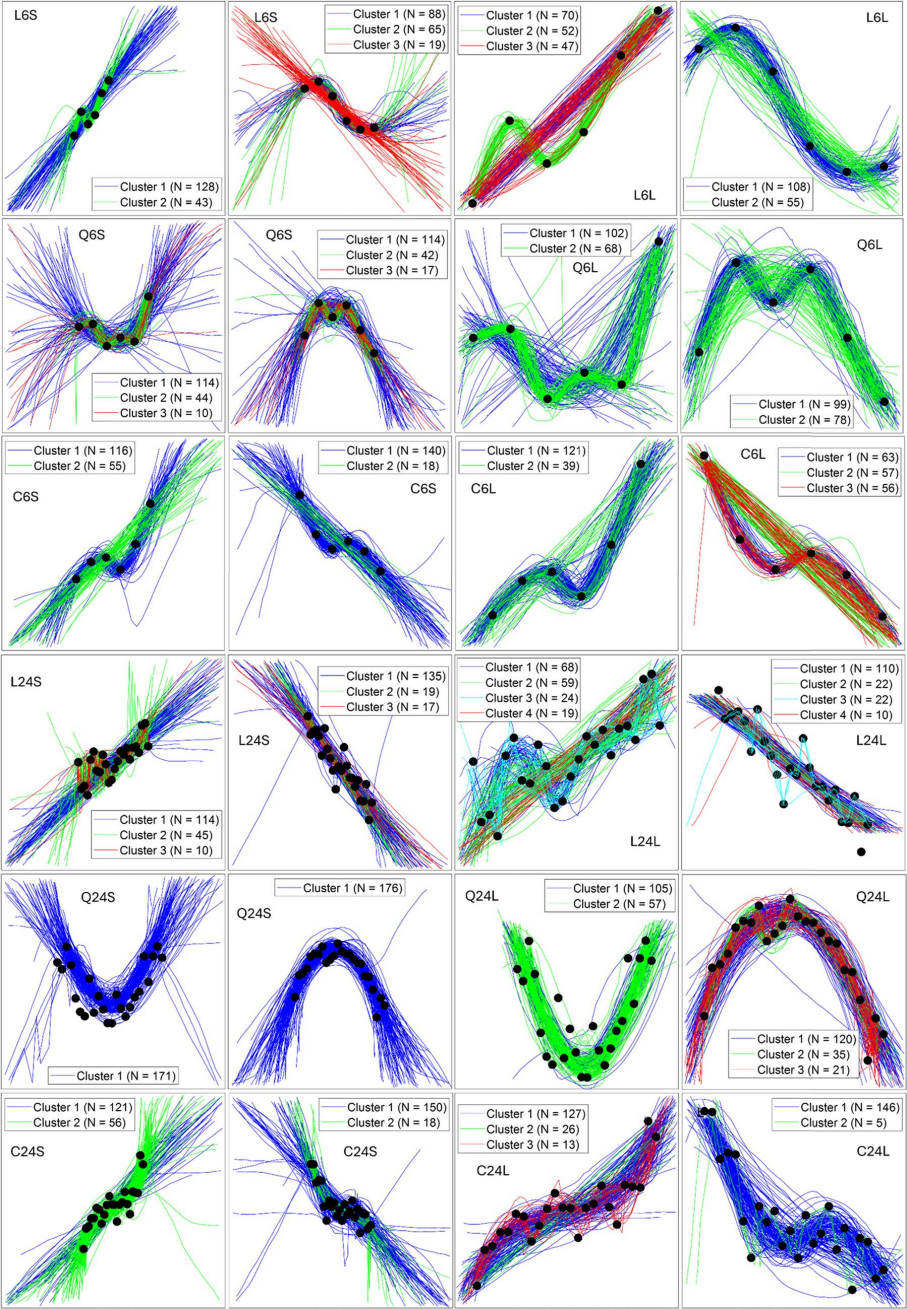
Observed response functions. The black dots show the scatterplot which was presented to participants. Each line shows a function that was drawn by participants. We colour-code the lines based on the outcome of our clustering analysis (see text for details). Note that only clusters with greater than 10 participants are shown. Problems are indexed by Generating function (L = Linear, Q = Quadratic, C = Cubic), Number of data points, and Scale (S = Small, L = Large)

The presented data and hand-drawn functions are displayed in Fig. 3. The panels in this figure show the presented data along with the individual functions generated by each participant. We color-coded the functions according to our cluster analysis results. The different clusters of data shown in the panels were determined by first quantifying the functional form of the response using GPR (Rasmussen & Williams, 2006), then clustering the estimated parameters using a nonparametric Bayesian clustering analysis (Navarro et al., 2006). Consequently, the clusters represent qualitatively different types of functions estimated as generating functions for the presented data. Clear individual differences are evident from the figure; however, the degree of individual differences varied between the functions. For instance, the quadratic functions in row 5 of Fig. 3 exhibit only *quantitative variation*; the basic form of the response function remains the same for all participants. By contrast, there is substantial qualitative variation in the function immediately above and below (in rows 4 and 6). To infer differences between clusters, we follow a functional analysis approach by summarizing each function by its parameters and analysing those parameters using an ANOVA (Ramsay & Silverman, 1997).

### Influence of amount of data, scale, and generating function

**Fig. 4:**
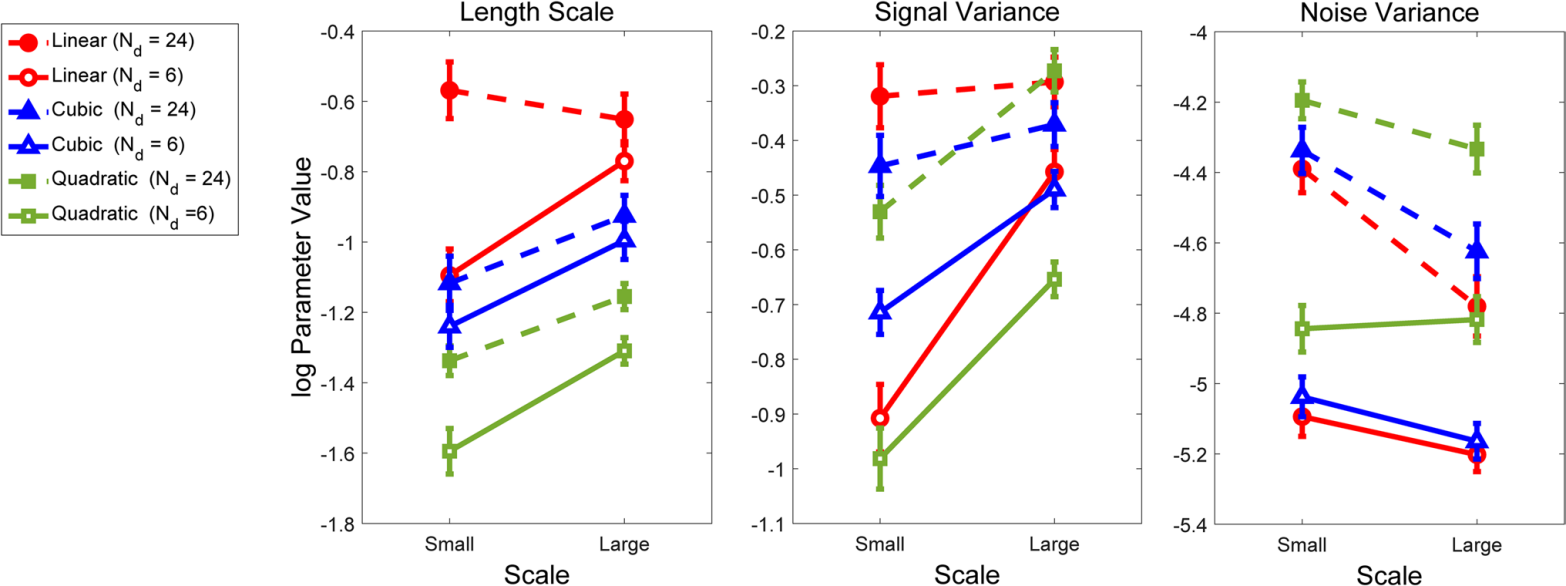
Average log hyperparameter values (Length Scale, Signal Variance, and Noise Variance) as a function of the scale of the data (small = zoomed in; large = zoomed out), number of data points, and generating function

We estimated the parameters of a GPR to each drawn function from each observer using maximum likelihood as described in the *Method* section. To examine the gross effect of changes in generating function, number of data points, and scale, we examined the overall average parameter values at each level of our manipulated factors. Figure 4 shows the average values of length scale, signal variance, and noise variance. The left hand panel shows the length scale parameter. There are three systematic effects evident in the length scale parameter: (1) larger presentation scales result in larger values of length scale (i.e., smoother functions; see Fig. 3); (2) increasing the number of data points results in larger length scales (i.e., smoother functions); and (3) there is a systematic ordering based on the generating functions. Linearly generated data elicit the largest length scales and, hence, the smoothest functions; quadratically generated data elicit small length scales, indicating a tendency toward data tracking for these functions, and cubically generated data fall in between the two. All main effects and interactions are significant, with the exception of the generating function $\times$ scale interaction; these results are presented in the Appendix Table 5.

The middle panel of Fig. 4 shows the results for the signal variance parameter. Here there two clear effects: (1) larger scales result in larger signal variance, and (2) smaller data sets have larger signal variance. The first effect is sensible, but the second effect is surprising. What the second effect suggests is that people “see” more of a signal when there are fewer data points. Combined with the length scale parameter estimates, this would indicate that when presented with fewer data points, people show more of a tendency to adopt data-tracking functions than when there are more data points. There are no clear effects of generating function; however, all main effects and interactions are significant (see Appendix Table 6).

Finally, the right-hand panel shows the noise variance parameter. Here are there are three clear effects: (1) linearly generated data have smaller noise variance than cubically generated data, which have smaller noise variance than quadratically generated data. (2) Noise variance tends to decrease at larger scales, but (3) noise variance increases with smaller data sets. All of these results are sensible. Again, all main effects and interactions are significant, with the exception of the number of data points, N_d,_ × generating function interaction and the three-way interaction between all factors (see Appendix Table 7). The influence of the generating function on the noise parameter suggests that with few data points, there is little to distinguish data generated from a noisy linear function and a noisy cubic function; both have the same global trend. Local fluctuations that differentiate a cubic function from a linear function should only arise at larger datasets. By contrast, a quadratic function may be evident at both small and large set sizes.

### Individual differences

In order to capture the individual differences across generated functions, we first applied a clustering algorithm to group the parameter data into different groups. This allows us to examine the main trends in the data as a function of the number of generating function, number of points, and scale. We additionally had expert raters classify each function (as linear, quadratic, or data tracking), and then using these classifications, we determined what values of the estimated parameters provided the maximum differentiation between function types. For example, can the length scale parameter alone differentiate between linear and data-tracking functions? Or does some combination of parameters provide a better classification between qualitatively different function types? Finally, we analysed the number of participants assigned to different groups for each stimulus and analysed the averaged parameter values for each group. The purpose of this analysis is to determine the average parameters that capture the key qualitative individual differences in the generated functions.

#### Benchmark classification data

Prior to examining the results of the clustering analysis, we first sought to provide some benchmark in order to understand how the parameters correspond to different types of functions (either globally influenced, polynomial-like functions or locally influenced, data-tracking functions). Preliminary examination of the Dirichlet process clusters revealed only clear groups of linear, quadratic, and data-tracking functions; consequently, for the expert-classified functions, we collapsed the cubic and data-tracking functions into a single category. We also removed the unclassifiable functions.

**Fig. 5:**
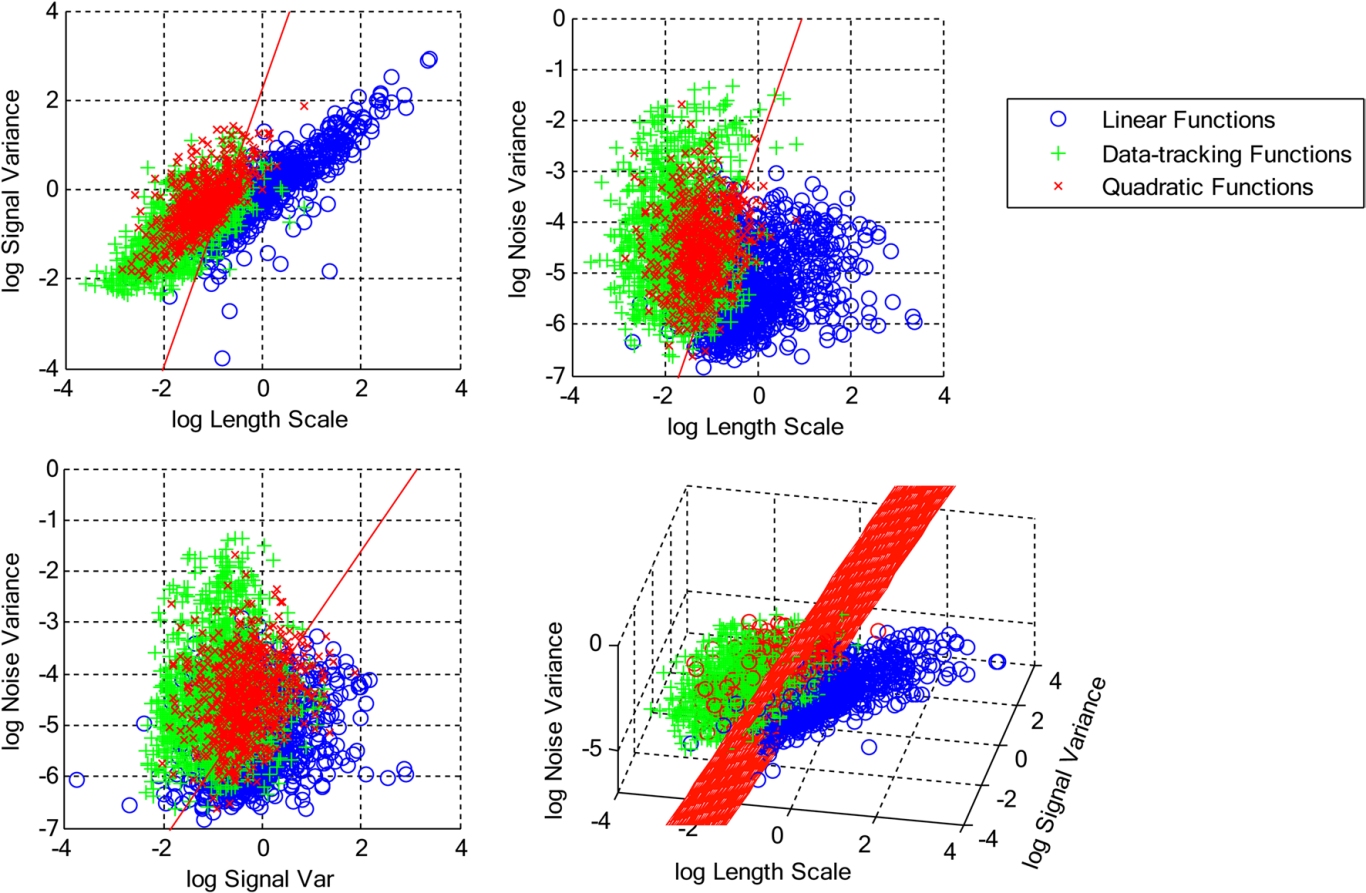
Each panel shows the best linear classifier applied to the expert-classified functions plotted as a function of log length scale and log signal variance hyperparameters (upper left panel), log length scale and log noise variance hyperparameters (upper right panel), log signal variance and log noise variance hyperparameters (lower left panel) and all three hyperparameters (lower right panel)

The purpose of this analysis was to show that the qualitative classifications of the functions (as determined by the expert classifications of linear, quadratic, or data-tracking) correspond to identifiable regions of the latent parameter space. Figure 5 shows the parameters of each function as estimated from the Gaussian process regression classified according to the expert. There is little distinction between the parameters of the functions classified as quadratic or data-tracking function; the parameters of these functions overlap almost completely. The functions classified as linear, however, are separated from the remaining parameters at least in some directions of the parameter space.

To determine how the parameter values map onto specific linear or data-tracking functions (and given the overlap, quadratic function), we fit a linear classifier to the three-dimensional parameter data. This linear plane is shown in the lower right-hand panel of Fig. 5; this classifier was able to separate the functions classified as linear from the functions classified as data tracking with 88.58% accuracy. To simplify the analysis, we also fit linear classifiers to each pair-wise set of parameters. All of the classifiers achieved reasonable accuracy; however, the two-dimensional classifier on log length scale $\times$ log noise variance achieved that same level of accuracy as the three-dimensional classifier (88.58%; the log length scale $\times$ log signal variance parameter had 86.88% accuracy and the log signal variance $\times$ log noise variance classifier had 80.13% accuracy). Consequently, we focus on the log length scale $\times$ log noise variance classifier (top right panel; see Fig. 5).

Based on this classifier, functions classified as linear tended to have smaller noise variance and larger length scales consistent with the intuition of how the parameters affect the generated function in Fig. 2. The key take-home message is that functions classified as data-tracking and quadratic tend to have smaller length scales and larger noise variance. (The boundary is given by -0.94 $\times$ log Length Scale + 0.35 $\times$ log Noise Variance + 0.91.)

The latent parameter space can be accurately divided according to the length scale and noise variance parameters. Based on the estimates of these two parameters, we can accurately determine whether the hand-drawn function is a linear polynomial function or whether the function tends to curve and “track” the local variations in the stimulus.

### Dirichlet process clustering analysis

The number of clusters found for each problem based on the Dirichlet process clustering analysis is shown in Table 1 (a subset of these is shown in each panel of Fig. 3). The average parameter values for the linear, quadratic and cubic functions are shown in Tables 2, 3, and 4, respectively. Some insight can be gained by analysing the number of clusters with respect to the properties of each of the functions. We conducted a 3 generating function × 2 number of data points × 2 scale ANOVA on the number of clusters and found a significant main effect of scale, *F*(1,12) = 6.15, *p* = 0.029, and a significant number of data points × scale interaction, *F*(1,12) = 6.15, *p* = 0.029 (note because the number of clusters is a discrete measure, these F-ratios are identical). No other effects were found. These results indicate that there is more qualitative variation across participants in responding (i.e., more clusters indicating different types of generated fucntions) with larger scales. Furthermore, the difference in the number of clusters between small and large scales is greater with a larger number of data points.

**Table 1:** Each entry provides the number of participants grouped into that cluster for each specific stimulus

	Cluster							
Problem	1	2	3	4	5	6	7	8
L6S	128	43	5	1				
L6S	88	65	19	2	2	1		
L6L	70	52	47	4	2	2		
L6L	108	55	6	4	2	2		
L24S	114	45	10					
L24S	135	19	17	3	2	1		
L24L	68	59	24	19	4	2	1	
L24L	110	22	22	11	7	2	2	1
Q6S	114	44	10	5	2	2		
Q6S	114	42	17	2	2			
Q6L	102	68	3	2	2			
Q6L	99	78						
Q24S	171	4	2					
Q24S	176	1						
Q24L	105	57	8	4	2	1		
Q24L	120	35	21	1				
C6S	116	55	4	2				
C6S	140	18	16	2	1			
C6L	121	39	9	4	2	1	1	
C6L	63	57	56	1				
C24S	121	56						
C24S	150	18	7	2				
C24L	127	26	13	8	3			
C24L	146	15	5	5	2	2	2	

Problems are indexed by Generating function (L = Linear, Q = Quadratic, C = Cubic), Number of data points, and Scale (S = Small, L = Large)

**Table 2:** Clusters for linearly generated functions. Each cell shows the mean (and standard deviation) of each parameter

		Cluster							
Problem	Parameter	1	2	3	4	5	6	7	8
L6S	Length Scale	0.68 (2.06)	0.09 (1.63)	0.50 (1.24)	0.15 (1.00)				
	Signal Var	0.72 (1.80)	0.26 (1.61)	0.75 (1.10)	1.96 (1.00)				
	Noise Var	0.01 (1.99)	0.01 (1.74)	0.04 (1.09)	0.01 (1.00)				
L6S	Length Scale	0.16 (1.41)	0.59 (1.80)	0.22 (1.33)	1.00 (1.00)	6.04 (2.10)	0.28 (1.00)		
	Signal Var	0.15 (1.20)	0.52 (1.78)	0.50 (1.76)	1.00 (1.00)	3.54 (2.00)	0.21 (1.00)		
	Noise Var	0.01 (1.71)	0.00 (1.75)	0.01 (1.46)	1.00 (1.00)	0.01 (1.13)	0.19 (1.00)		
L6L	Length Scale	0.65 (1.38)	0.87 (2.25)	0.22 (1.26)	0.30 (1.51)	29.25 (1.04)	0.56 (2.07)		
	Signal Var	0.71 (1.21)	1.13 (1.75)	0.52 (1.17)	1.29 (1.25)	18.13 (1.03)	0.67 (1.58)		
	Noise Var	0.00 (1.38)	0.00 (1.50)	0.01 (1.61)	0.01 (1.34)	0.00 (1.08)	0.03 (1.68)		
L6L	Length Scale	0.32 (1.37)	0.56 (1.64)	0.60 (1.05)	0.97 (7.16)	0.29 (1.03)	0.48 (2.80)		
	Signal Var	0.41 (1.12)	0.67 (1.54)	0.57 (1.08)	1.15 (2.78)	1.33 (1.01)	1.75 (2.20)		
	Noise Var	0.01 (1.68)	0.00 (1.54)	0.02 (1.15)	0.01 (4.68)	0.03 (1.35)	0.08 (34.29)		
L24S	Length Scale	0.88 (2.79)	0.18 (2.25)	0.43 (1.41)					
	Signal Var	0.75 (2.28)	0.41 (2.06)	0.33 (1.64)					
	Noise Var	0.01 (1.76)	0.02 (3.18)	0.11 (1.48)					
L24S	Length Scale	0.41 (1.90)	3.22 (1.57)	1.92 (1.28)	0.56 (1.36)	0.43 (1.84)	1.00 (1.00)		
	Signal Var	0.69 (1.47)	3.06 (1.66)	1.48 (1.14)	0.43 (1.61)	2.58 (1.30)	1.00 (1.00)		
	Noise Var	0.01 (2.04)	0.01 (1.64)	0.02 (1.34)	0.16 (1.97)	0.01 (3.10)	1.00 (1.00)		
L24L	Length Scale	0.49 (1.80)	0.20 (1.45)	1.57 (1.63)	0.58 (7.39)	0.24 (1.81)	0.64 (1.88)	1.74 (1.00)	
	Signal Var	0.77 (1.64)	0.41 (1.29)	1.17 (1.44)	0.79 (2.98)	0.27 (1.29)	0.74 (1.52)	0.48 (1.00)	
	Noise Var	0.00 (1.89)	0.01 (1.69)	0.01 (1.66)	0.03 (3.89)	0.18 (1.04)	0.52 (2.50)	0.21 (1.00)	
L24L	Length Scale	0.53 (1.51)	2.80 (1.53)	0.36 (1.93)	0.46 (3.82)	0.95 (1.45)	0.60 (1.18)	0.60 (2.13)	4.49 (1.00)
	Signal Var	0.72 (1.32)	2.16 (1.58)	0.64 (1.35)	1.07 (2.04)	0.80 (1.42)	1.62 (1.06)	2.72 (1.48)	1.59 (1.00)
	Noise Var	0.00 (1.56)	0.01 (1.55)	0.02 (1.94)	0.03 (5.64)	0.23 (1.36)	0.01 (2.99)	0.01 (2.58)	0.00 (1.00)

Problems are indexed by Generating function (L = Linear, Q = Quadratic, C = Cubic), Number of data points, and Scale (S = Small, L = Large)

**Table 3:** Clusters for quadratically generated functions. Each cell shows the mean (and standard deviation) of each parameter

		Cluster							
Problem	Parameter	1	2	3	4	5	6	7	8
Q6S	Length Scale	0.25 (1.77)	0.07 (1.30)	0.31 (1.23)	3.36 (1.40)	2.65 (1.61)	2.08 (2.49)		
	Signal Var	0.43 (1.94)	0.14 (1.23)	0.52 (1.24)	1.30 (1.75)	0.54 (1.49)	0.22 (1.56)		
	Noise Var	0.01 (2.14)	0.01 (2.12)	0.03 (1.35)	0.00 (1.62)	0.01 (1.39)	0.05 (56.02)		
Q6S	Length Scale	0.23 (1.73)	0.08 (1.28)	0.41 (1.32)	4.42 (1.19)	0.67 (1.77)			
	Signal Var	0.53 (1.67)	0.19 (1.29)	0.57 (1.34)	2.47 (1.01)	0.15 (14.44)			
	Noise Var	0.01 (2.08)	0.01 (1.83)	0.03 (1.41)	0.01 (1.94)	0.05 (72.31)			
Q6L	Length Scale	0.35 (1.65)	0.18 (1.25)	0.36 (2.23)	1.00 (1.00)	0.25 (1.12)			
	Signal Var	0.53 (1.54)	0.42 (1.14)	0.88 (5.44)	1.00 (1.00)	0.79 (1.06)			
	Noise Var	0.00 (1.92)	0.02 (2.00)	0.03 (17.29)	1.00 (1.00)	0.06 (1.20)			
Q6L	Length Scale	0.21 (1.25)	0.38 (1.65)						
	Signal Var	0.45 (1.17)	0.70 (1.68)						
	Noise Var	0.01 (1.71)	0.00 (1.96)						
Q24S	Length Scale	0.27 (1.77)	0.07 (1.11)	0.27 (1.15)					
	Signal Var	0.56 (1.90)	0.18 (1.19)	2.13 (1.59)					
	Noise Var	0.01 (2.00)	0.06 (1.51)	0.01 (1.17)					
Q24S	Length Scale	0.26 (1.69)	1.00 (1.00)						
	Signal Var	0.63 (1.83)	1.00 (1.00)						
	Noise Var	0.02 (1.92)	1.00 (1.00)						
Q24L	Length Scale	0.33 (1.53)	0.25 (1.29)	0.13 (1.23)	0.28 (1.52)	1.00 (1.00)	2.58 (1.00)		
	Signal Var	0.95 (1.53)	0.57 (1.14)	0.48 (1.15)	0.50 (1.34)	1.00 (1.00)	2.46 (1.00)		
	Noise Var	0.01 (1.88)	0.01 (1.57)	0.08 (1.80)	0.22 (1.11)	1.00 (1.00)	0.00 (1.00)		
Q24L	Length Scale	0.37 (1.60)	0.38 (1.50)	0.17 (1.18)	2.31 (1.00)				
	Signal Var	0.70 (1.57)	1.23 (1.92)	0.44 (1.14)	6.51 (1.00)				
	Noise Var	0.01 (1.78)	0.04 (2.50)	0.03 (1.46)	0.02 (1.00)				

Problems are indexed by Generating function (L = Linear, Q = Quadratic, C = Cubic), Number of data points, and Scale (S = Small, L = Large)

**Table 4:** Clusters for cubically generated functions. Each cell shows the mean (and standard deviation) of each parameter

		Cluster							
Problem	Parameter	1	2	3	4	5	6	7	8
C6S	Length Scale	0.16 (1.46)	0.70 (2.12)	0.54 (1.22)	0.20 (1.24)				
	Signal Var	0.37 (1.48)	0.75 (1.77)	0.56 (1.19)	1.42 (1.26)				
	Noise Var	0.01 (1.86)	0.00 (1.52)	0.01 (1.19)	0.01 (2.88)				
C6S	Length Scale	0.29 (1.97)	0.51 (1.24)	0.37 (1.76)	1.00 (1.00)	8.13 (1.00)			
	Signal Var	0.48 (1.57)	0.48 (1.21)	0.85 (1.53)	1.00 (1.00)	5.85 (1.00)			
	Noise Var	0.01 (2.25)	0.00 (1.45)	0.01 (1.40)	1.00 (1.00)	0.01 (1.00)			
C6L	Length Scale	0.23 (1.29)	0.63 (2.06)	0.41 (1.31)	6.09 (1.44)	0.56 (3.14)	1.01 (1.00)	1.42 (1.00)	
	Signal Var	0.49 (1.17)	0.92 (1.43)	0.49 (1.17)	3.06 (1.33)	1.01 (1.51)	0.89 (1.00)	3.57 (1.00)	
	Noise Var	0.01 (1.64)	0.01 (2.52)	0.00 (1.23)	0.01 (1.08)	0.10 (4.41)	0.01 (1.00)	0.01 (1.00)	
C6L	Length Scale	0.49 (1.44)	0.24 (1.27)	0.56 (2.17)	13.61 (1.00)				
	Signal Var	0.60 (1.21)	0.46 (1.17)	0.87 (1.47)	12.54 (1.00)				
	Noise Var	0.00 (1.33)	0.01 (1.44)	0.00 (1.81)	0.00 (1.00)				
C24S	Length Scale	0.22 (2.02)	1.17 (2.58)						
	Signal Var	0.68 (1.80)	1.11 (2.19)						
	Noise Var	0.02 (2.31)	0.01 (1.93)						
C24S	Length Scale	0.24 (2.09)	0.36 (1.65)	4.17 (2.08)	1.20 (1.29)				
	Signal Var	0.42 (1.68)	1.42 (1.68)	3.00 (2.09)	0.44 (3.23)				
	Noise Var	0.01 (2.23)	0.03 (2.22)	0.01 (1.44)	0.07 (45.56)				
C24L	Length Scale	0.46 (1.72)	0.18 (1.44)	5.13 (1.86)	0.63 (1.36)	0.81 (2.61)			
	Signal Var	0.82 (1.64)	0.40 (1.14)	2.28 (2.01)	0.77 (1.16)	0.43 (1.60)			
	Noise Var	0.01 (1.98)	0.02 (1.79)	0.01 (1.91)	0.10 (1.18)	0.12 (1.42)			
C24L	Length Scale	0.31 (1.55)	0.33 (1.35)	0.13 (1.15)	0.65 (1.14)	3.25 (1.33)	0.45 (1.62)	0.67 (1.77)	
	Signal Var	0.58 (1.33)	0.51 (1.18)	0.49 (1.16)	1.68 (1.11)	2.91 (1.28)	1.65 (1.64)	1.77 (2.25)	
	Noise Var	0.01 (1.90)	0.12 (1.54)	0.08 (1.70)	0.01 (2.56)	0.00 (1.17)	0.00 (1.61)	0.10 (26.56)	

Problems are indexed by Generating function (L = Linear, C = Cuadratic, C = Cubic), Number of data points, and Scale (S = Small, L = Large)

Having linked specific parameter values with specific types of functions using the hand-rated expert analysis, we can now interpret the clusters presented in Tables 2, 3 and 4. Careful observation of Fig. 3 shows that not all qualitatively similar functions are classified into the same qualitative group. For instance, in the third column of row 1 (problem L6L), a few “data-tracking” functions classified into Cluster 2 are qualitatively similar to functions in Cluster 3. The reason for this classification is twofold: first, the clustering analysis is probabilistic and Cluster 2 has more data points than Cluster 3. Recall that the prior over cluster assignment favours larger groups. Second, the functions in each cluster are also determined by the signal and noise variance parameters, whose effects are not directly observable in the figure. For this second reason, it is also important to note that not all clusters appear qualitatively different (i.e., in Fig. 3). For instance, in problem L6L, Cluster 1 and Cluster 2 in the top left panel have functions that are qualitatively similar; however, Cluster 2 on average has a more variable signal variance distribution.

Nevertheless, Fig. 3 makes it clear that there are considerable individual differences that range from quantitative variation in the exact shape of a function (even though all individuals adopt qualitatively similar functions; e.g., fifth row, second column, problem Q24S of Fig. 3) to considerable qualitative variations. These qualitative variations look to the eye to be consistent with functions that tend to either follow global trends in the data resulting in smooth polynomial-like functions to functions that move up and down with each local fluctuation in the data. Both types are adequately captured by our flexible GPR using the squared exponential kernel.

Across all participants and trials, length scale is positively correlated with signal variance (r = 0.76, p < 0.001) and negatively correlated with noise variance (r = -0.14, p < 0.001). Noise variance and signal variance are uncorrelated (r = 0.02, p = 0.19). As shown in Fig. 3, signal variance and noise variance both play a large role in moderating the effect of variation in length scale; consequently, these correlations are not surprising. To examine the stability of the individual differences across different function trials, we computed the average pairwise correlation between the estimated parameters for each trial. For all parameters, the average correlation was significantly greater than 0, indicating that along with the effects of specific displays, response functions are influenced by a consistent individual bias toward either more global or more local functions. (Length scale, average *r* = 0.23, *t*(275) = 36.12, *p* < 0.001; signal variance, average *r* = 0.21, *t*(275) = 30.73, *p* < 0.001; noise variance, average *r* = 0.21, *t*(275) = 35.41, *p* < 0.001.)

## Discussion

In summary, using an open-ended function-estimation drawing task, we show that there are individual differences in the function that university students generate, with some observers preferring functions that utilize global aspects of the presented data, and consequently, produce functions that are more polynomial-like, and others who focus on local aspects of the data, and produce functions that tend to “track” the presented data. We have provided a detailed explanation of Gaussian process regression (GPR), which we used to capture these individual differences. We also applied a non-parametric Bayesian clustering analyses (i.e., Dirichlet-process clustering) to provide more compelling summary of what the Gaussian process parameters “mean” in terms of the types of functions that the parameters describe.

There are several plausible psychological explanations for these individual differences. The first is that individuals may attend to different aspects of the scatterplots, which in turn results in different inferences about the underlying generating function. Scatterplots, such as the displays used in this study, are multidimensional (Cleveland et al., 1982; Collyer et al., 1990), and given the same data, different individual may focus on different dimensions of a scatterplot resulting in different inferences about the underlying function. This tendency seems to be influenced by the specific features of the graph. For instance, quadratically generated data presented at a small scale seems to result in consistent quadratic functions across participants. By contrast, graphs with small numbers of points presented at large scale seem to result in more variation between groups of participants (e.g., problem L6L in Fig. 3).

A second plausible explanation is that increased experience with mathematical functions may lead to more polynomial-like functions since greater mathematical experience may improve comprehension of function estimation problems. We note that this experience is likely highly mixed in our sample. However, it is also worth noting that in our initial pilot tests with psychology graduate students, all of whom presumably had some experience with statistical methods and analysis, the individual differences were still apparent. At any rate, in going beyond previous studies of contour interpolation, collinearity perception, and correlation judgement by using a variety of different functions, we have uncovered a novel method of tapping into a relatively consistent difference in how people infer and generate functions for noisy data.

Notwithstanding the evident individual differences, some general inferences can be drawn about the influence of specific aspects of the display on the estimated functions. For instance, larger presentation scales, increased number of data points, and underlying generating functions of a lower-polynomial order result in smoother generated functions on average. Conversely, our sample tended to generate functions that track the data when there are fewer presented data points. This is interesting since the additional complexity of the data-tracking function is not supported by the smaller number of points. Such a bias is consistent with the study of contour interpolation, which shows that with small numbers of data points, only points nearest to the region of interpolation matter (Hon et al., 1997). Our results suggest that this is also true for the case where considerable noise is added to the data, but that this influence of local points is overcome with the addition of more data.

We further see two broader implications from our analysis. The first is that our drawing methodology may provide a relatively pure way of examining aspects of quantitative causal reasoning. This might allow for a fuller exploration of individual differences in causal explanation. The second is that these results may have practical relevance for science communication. If researchers have a clear sense of the underlying generating function of a dataset, then communicating that function graphically (i.e., drawing the generating function as part of the scatter plot) will facilitate communication by fostering a common representation among observers.

To fully generalize our findings, further testing with a more general sample is needed. That work should also fully survey prior experience with mathematical functions in order to determine the possible cause of the individual differences.

## Data Availability

Data and questionnaire are available at: https://github.com/knowlabUnimelb/FUNCTION_ESTIMATION.

## Appendix

**Table 5:** Results of a 2 number of data points × 3 generating function × 2 scale repeated-measures ANOVA on the estimated length scale parameters

Effect	df_Between_	df_Within_	F	MSE	p	η_p_^2^
Number of data points (ND)	1	176	65.63	0.35	< 0.001	0.27
Generating Function (GF)	2	352	185.79	0.32	< 0.001	0.51
Scale (S)	1	176	41.06	0.47	< 0.001	0.19
ND × GF	2	352	9.24	0.25	< 0.001	0.05
ND × S	1	176	18.06	0.25	< 0.001	0.09
GF × S*	1.93	338.81	3.23	0.23	0.04	0.02
ND × GF × S*	1.93	340.03	7.78	0.22	0.001	0.04

* Greenhouse–Geisser correction is reported due to a violation of the sphericity assumption

df = degrees of freedom, MSE = mean square error, η_p_^2^ = partial eta squared

**Table 6:** Results of a 2 number of data points × 3 generating function × 2 scale repeated measures ANOVA on the estimated signal variance parameters

Effect	df_Between_	df_Within_	F	MSE	p	η_p_^2^
Number of data points (ND)	1	176	316.06	0.18	< 0.001	0.09
Generating Function (GF)	2	352	17.80	0.16	< 0.001	0.09
Scale (S)	1	176	74.49	0.36	< 0.001	0.30
ND × GF	2	352	18.33	0.14	< 0.001	0.09
ND × S	1	176	50.53	0.12	< 0.001	0.22
GF × S	2	352	8.28	0.11	< 0.001	0.05
ND × GF × S	2	352	12.62	0.12	< 0.001	0.06

df = degrees of freedom, MSE = mean square error, η_p_^2^ = partial eta squared.

**Table 7:** Results of a 2 number of data points × 3 generating function × 2 scale repeated measures ANOVA on the estimated noise variance parameters

Effect	df_Between_	df_Within_	F	MSE	p	η_p_^2^
Number of data points (ND)	1	176	313.58	0.57	< 0.001	0.64
Generating Function (GF)	2	352	65.28	0.30	< 0.001	0.27
Scale (S)	1	176	43.20	0.38	< 0.001	0.20
ND × GF*	1.84	323.59	0.62	-	0.53	-
ND × S	1	176	14.49	0.33	< 0.001	0.08
GF × S*	1.89	333.28	7.39	0.34	0.001	0.04
ND × GF × S	2	352	1.12	-	0.33	-

* Greenhouse–Geisser correction is reported due to a violation of the sphericity assumption

df = degrees of freedom, MSE = Mean Square Error, η_p_^2^ = partial eta squared.

## Code availability

Code is available at: https://github.com/knowlabUnimelb/FUNCTION_ESTIMATION.

## Declarations

### Open practices statement

All data and materials are available at https://github.com/knowlabUnimelb/FUNCTION_ESTIMATION

The experiments were not preregistered.
